# New York Letter

**Published:** 1898-09

**Authors:** Chas. J. Proben

**Affiliations:** 970 Lexington Ave., New York City


### *NEW YORK LETTER.*

---

NEW YORK, August, 1898.

*To the Editor of the Atlanta Medical and Surgical Journal:*

Reports of proceedings of some of our medical societies so frequently bring up for consideration the feasibility of adopting operative procedure, when the question whether uncontroverted facts as alleged should really be accepted as indicative of such interference. Such statements frequently go unchallenged without even a dissenting voice. On the face of these publications it appears evident that they savor of unguarded pretensions of empiricism, bordering on pure unalloyed charlatanry. Quite frequently reports of some of our prominent scientific societies contain descriptions of operation, not in any sense minor, which appear brazenly contraindicated, but are simply extolled by an unscrupulous and deceiving tongue simply to create an impression upon the audience. Probably in no domain of medicine during the last decade has more rapid strides been made than in surgery, especially is it so with gynecology; and verily it may be said that in no branch has a greater tendency manifested itself to resort to ambiguous operations, and in no branch has this been more abused than in gynecology. Indiscriminately our gynecologists seek to outdo each other and record novel procedures and a large number of operations, without in any way innately consulting the future welfare of the patient. This may appear ludicrous, but it is true. Were it not for the fact that this condition of affairs constantly tends to increase and assume an alarming aspect, I should never have tried to influence or curtail the operative furor of a few of my friends. Only during the past week two similar cases have come under my observation showing the mutilating effects of the scalpel guided by a morbid brain. One of these cases is so pathetic that I cannot refrain from referring to it, but cursorily. During my summer vacation I left a lovely young woman who was wearing a pessary, inserted for a plain, uncomplicated case of retroversion, in charge of one of my confrères to look after. Of the latter condition I was fairly certain that I was correct. This patient, however, was informed by a friend that a certain gynecologist could cure her quickly, and she induced her to consult him. He reiterated that a pessary was of no avail, and induced her to submit to an operation, to which she consented, and following which he guaranteed complete relief to all symptoms and a permanent cure. What the operation was I do not know, but the result was that this brilliant operation made the patient worse, terminating finally in another operation, with a guarantee affixed that she would fully recover after total extirpation of the uterus. I am well informed of the facts of the case, and never should I have been induced to raise my pen in protest were it not that my passion was infuriated at the reckless disregard, aye haughtiness, with which this practitioner viewed his destructive mutilating work. At the expense of these poor creatures, sacrificing as much tissue as possible, repeated successes make these brazen-faced men bolder and bolder, and the furor to equip or excel each other is so great that poor suffering humanity is their goal. Yes, by success they mean quite a different thing from ultimate relief of suffering. Success is gauged only by their avaricious self-aggrandizement. I am quite sure I voice the sentiments of a large number of our profession when I say that almost at the eve of this century competition and the struggle for a livelihood have become more acute, and the energetic young man is taught by the energetic older members those unscrupulous attainments and practices in order to aggrandize oneself and perform uncalled-for operations at the expense and sacrifice of some poor, inert, harmless organ. Thus its victims strut about in daily intercourse with the brilliant physician who points with pride to his wobegone fame. The wonderful successes attained at a great cost are constantly ventilated; the great number of brilliant operations give vent to expressions of the acquisition of a grand and noble man to the community, and a shining light has his beavers secretly and quietly working for his salvation, the quintessence of which is merely to him of a pecuniary character. Proud and inflated, he considers himself as a distinct entity from all of the rest, and is ready to explode at any minute; but in his own consciousness he well knows that his fondness for ostentation is forgotten at the expense of some poor creature, and deceptive pretensions merely seek to induce a general plethora of his pocket-book rather than a scrupulous pathetic obligation to his patient. The relation he should bear to his patients seeks a remunerative outlet rather than to succor and alleviate. The results appear unmistakably evanescent, rather than of permanent benefit, but with this he has not time to be annoyed. This glaring evil is especially linked to our larger cities, which are apt to breed embryo instructors and professors with utter disregard of a clear conscience. Such heinous and ghastly procedures as I have depicted—and I really speak reservedly, not condemnatory—are of almost daily occurrence, and afford a subject for just criticism. How different is the aspect of the question when propounded to them if they were put face to face and requested to perform such operations upon the members of their own household. Record of a large number of such operations appear brilliant; paper is fortunately submissive, and, usually, so is the profession; what consternation would it create if it could only speak the result! Frequently it dawns upon me that the true, veritable physician is gradually passing away; as the century draws to a close the weak must make room for the most corrupt, the most unscrupulous, and the most degenerate. Regardless of the future welfare of their patients, if these physicians were confronted with the result of their over-zealous activity years thereafter, what an array of failures this would depict! and how their consciences, if any, would compel them to crawl and cower like a dog under the lash of a whip! The pangs of their guilt would be manifestly visible upon their faces. Mr. Editor, you probably will have considered me rather severe in my criticisms of this class, but it is only an iota of what they justly deserve. They should be actuated by nobler motives of charity, and embody those true attributes requisite to the vocation they have selected. This result is partly brought about by egotism, the greed for self-aggrandizement and the strife to excel others, and they consider themselves of a higher ideal, and believe with Dr. Stockton, who says "the majority are always wrong." You will agree with me the case I have but referred to is a pathetic one. Similar ones occurring daily in a large metropolis like ours, where aphobia for operations walks extant and can be easily acquired. One can always find one of those charming, progressive members of our profession who is satisfied to operate in order simply to quiet a person's fears, as they say, when absolutely no indication for any operative interference exists. A cure is certainly promised, but what are we to consider a cure? If an innocent and harmless organ is slightly bent out of its natural place without producing scarcely any symptoms at all, radicalism suggests to them the only way is to unsex the woman, not in order to help her, but to extol their wonderful boldness and achievements at a medical meeting; to circulate reports among the patients' numerous friends; to obtain prestige, and to gloat over their success--all strictly within our rigid code of ethics. But not for a moment would this man dare to divulge to his patient that she would practically become unsexed, make her unfit for maternity, and deprive her of the greatest charm God had given her. Unable to beget children, the aim of life is practically at an end. With a promise to relieve her symptoms, they now really appear and increase in number, and a variety of phenomena call for explanation. Providing our friend tries to be scrupulous, his conscience will rebel, and he must exert his energy by attacking other organs. This operation was successful; why should not the others also be so. The future of motherhood was destroyed, now you might just try and go a step further, and wantonly remove the vagina, and attack other organs; you will either hit or miss. With impunity you can fortify your conscience, actuated, of course, only by motives of charity, and relieve suffering humanity by performing another operation on the same poor creature. Go right ahead and be bold. No guilt but diffidence and pride attaches to you. With impunity you have attacked, relieved (?) and removed the burdens of suffering humanity. You are an up-to-date practitioner, and if you do not soon amend you must grasp the last straw and sink to the bottom, with scalpel in hand, embodying destruction and warfare against all organs that permit of removal. Max Nordau has deplorably overlooked you as a class whose charitable instincts are worthily humane in one extreme, and that is the other. (?) But, you may argue, this science is only in its infancy and requires encouragement. True, it has brighter hopes, or, rather, brighter hopes may be expected in the future. Poor, charitable man! how sympathetic you appear in the guise of a learned and true physician in order to relieve your fellow men of their burdens (of money), but I tell you the hereafter will not be able to cope with those just burdens you will duly be required to carry. You pretend to be ignorant of the reason why, but the fruits of your worldly and badly-begotten labors are not so joyfully enjoyed as you appear to manifest. You have often tasted the scorpion's sting, but have been immune to its venomous effects, as your callousness has been pretty well cultivated. How sweet, after all, it is to bear another's burden! and what a consolation you appear to derive from it all!

The world is all a fleeting show,
For man's illusion given;
The smiles of joy, the tears of woe,
Deceitful shine, deceitful flow,
There's nothing true but Heaven!—*Moore.*

Pardon my insistency, Mr. Editor, but am I right?

I am your obedient servant,

CHAS. J. PROBEN, M.D.

*970 Lexington Ave., New York City.*

---

### TREATMENT OF WHOOPING-COUGH.

Marfan (*Lyon médical*, June 5th) recommends the following:

| | | |
|---|---|---|
| ℞ | Antipyrin | 15 grains. |
| | Syrup of belladonna | 6 drachms. |
| | Syrup of Tolu | 3 ounces. |

The initial daily amount recommended to be taken is—from birth to one year, from one to two teaspoonfuls; from two to three years, four teaspoonfuls; from five to ten years, six teaspoonfuls. The dose should be augmented daily by half a teaspoonful until either a diminution of the paroxysms occurs or until intolerance is reached, as evidenced by dilatation of the pupil, dryness of the throat, etc.—*N. Y. Med. Jour.*

